# Reawakening of a dormant diaphragmatic hernia: a case of delayed post-traumatic presentation

**DOI:** 10.1093/jscr/rjaf705

**Published:** 2025-09-06

**Authors:** Nang Van Pham, Tuan Van Nguyen, Doi Van Mai, Phu Diep Thien Duong, Kiet Gia Le Nguyen, Huan Hoang Lam, Jan Willem Greve

**Affiliations:** Department of General Surgery, Can Tho University of Medicine and Pharmacy Hospital, 179 Nguyen Van Cu Street, Tan An Ward, Can Tho City 900000, Viet Nam; Department of General Surgery, Can Tho University of Medicine and Pharmacy, 179 Nguyen Van Cu Street, Tan An Ward, Can Tho City 900000, Viet Nam; Department of General Surgery, Can Tho University of Medicine and Pharmacy Hospital, 179 Nguyen Van Cu Street, Tan An Ward, Can Tho City 900000, Viet Nam; Department of General Surgery, Can Tho University of Medicine and Pharmacy, 179 Nguyen Van Cu Street, Tan An Ward, Can Tho City 900000, Viet Nam; Department of General Surgery, Can Tho University of Medicine and Pharmacy Hospital, 179 Nguyen Van Cu Street, Tan An Ward, Can Tho City 900000, Viet Nam; Department of General Surgery, Can Tho University of Medicine and Pharmacy, 179 Nguyen Van Cu Street, Tan An Ward, Can Tho City 900000, Viet Nam; Department of General Surgery, Can Tho University of Medicine and Pharmacy Hospital, 179 Nguyen Van Cu Street, Tan An Ward, Can Tho City 900000, Viet Nam; Department of General Surgery, Can Tho University of Medicine and Pharmacy Hospital, 179 Nguyen Van Cu Street, Tan An Ward, Can Tho City 900000, Viet Nam; Department of General Surgery, Can Tho University of Medicine and Pharmacy Hospital, 179 Nguyen Van Cu Street, Tan An Ward, Can Tho City 900000, Viet Nam; NUTRIM, Department of Surgery, Maastricht University, PO Box 616, 6200 MD Maastricht, Limburg, The Netherlands

**Keywords:** diaphragmatic hernia, trauma, laparoscopic repair, mesh

## Abstract

Traumatic diaphragmatic hernia (TDH) is a rare condition, especially on the right side, often lacking specific symptoms and sometimes manifesting years after the initial trauma. This case report highlights the importance of thorough history-taking, physical examination, and radiological imaging in the diagnosis and treatment of TDH. A 41-year-old male presented with recurrent epigastric pain and respiratory problems, initially treated for gastritis without relief. Further investigation revealed a large right-sided diaphragmatic hernia linked to a prior trauma. The patient underwent successful laparoscopic repair via an abdominal approach, with the defect closed using sutures and polytetrafluoroethylene mesh. Identifying delayed TDH is challenging, but a comprehensive diagnostic evaluation can ensure timely diagnosis and effective treatment, helping to prevent serious complications.

## Introduction

Traumatic diaphragmatic hernia (TDH) is uncommon, occurring in 0.8%–6% of blunt trauma cases and up to 17% in thoracoabdominal-penetrating trauma [1]. Left-sided hernias are more common (50%–80%) due to the protective effect of the liver, whereas right-sided hernias occur in 12%–40%. Bilateral cases are rare (1%–9%) [2, 3]. The pressure gradient between the thoracic and abdominal cavities and constant diaphragmatic motion hinder spontaneous healing, and when intra-abdominal pressure increases may enlarge existing defects [4].

Diagnosis of TDH can be challenging due to nonspecific symptoms and delayed presentation [5]. A detailed trauma history and appropriate imaging modalities are crucial.

## Case report

A 41-year-old man presented with a 2-month history of recurrent epigastric pain radiating to the right upper quadrant. The pain worsened on an empty stomach and during meals. He reported no bowel habit changes, vomiting, or weight loss. Despite self-medicating with antacids, he experienced no relief. Notably, he had sustained right-sided chest trauma in a motorcycle accident 20 years prior with rib fractures and a hemothorax, treated with pleural drainage. He recently developed dyspnea, particularly on deep inspiration and while sleeping.

**Figure 1:**
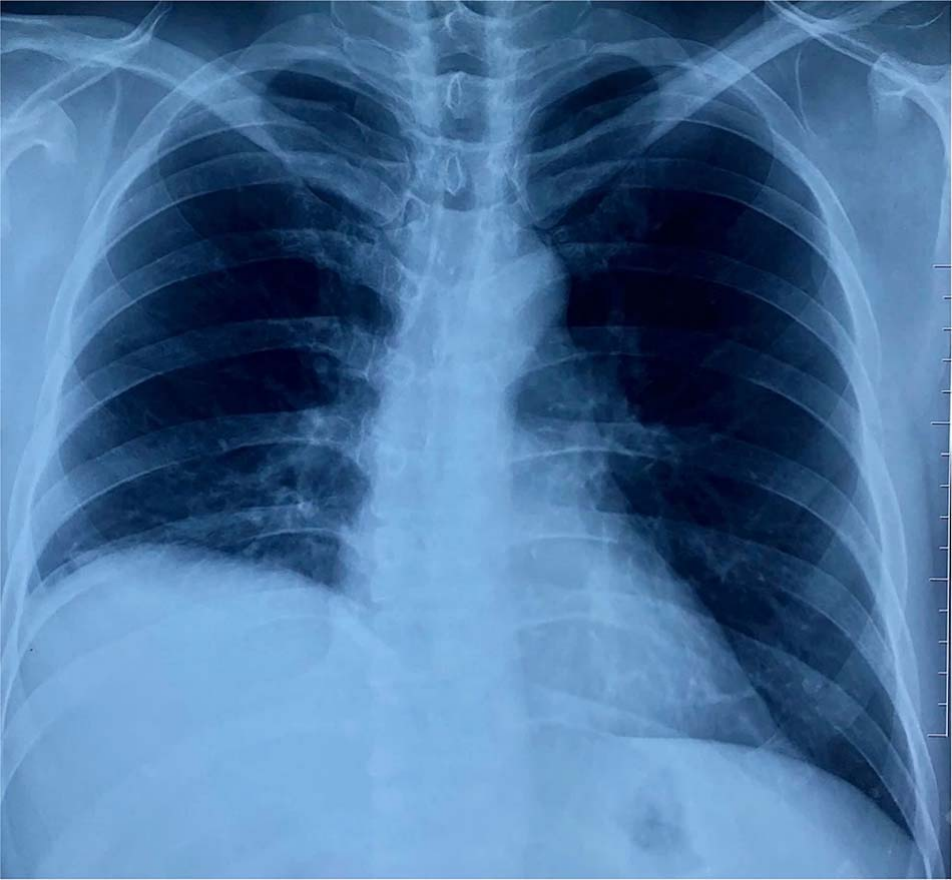
The chest X-ray demonstrated an elevated right hemidiaphragm.

**Figure 2:**
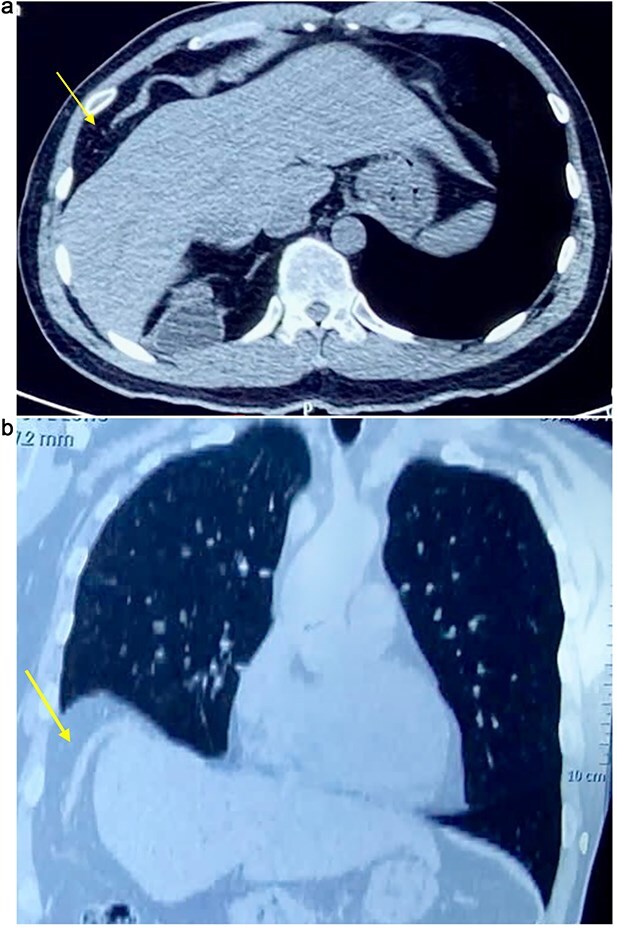
(a) Axial CT scan showing an arrow pointing to a discontinuity of the right diaphragm muscle near the right chest wall. (b) Transverse CT scan showing an arrow pointing to a communication between the peritoneal fat of the abdominal cavity and the right pleural space at the lung base.

On physical examination, vital signs were stable, and there were no abnormalities. Blood tests and abdominal ultrasound were normal. Gastroscopy shows no lesions. Chest X-ray shows an elevated right hemidiaphragm (Fig. 1). Computed tomography (CT) scan revealed a discontinuity of the right diaphragmatic muscle near the chest wall, with an ~9 × 5 cm hernia defect. This defect established communication between the peritoneal fat and the right pleural space at the lung base (Fig. 2a and b).

**Figure 3:**
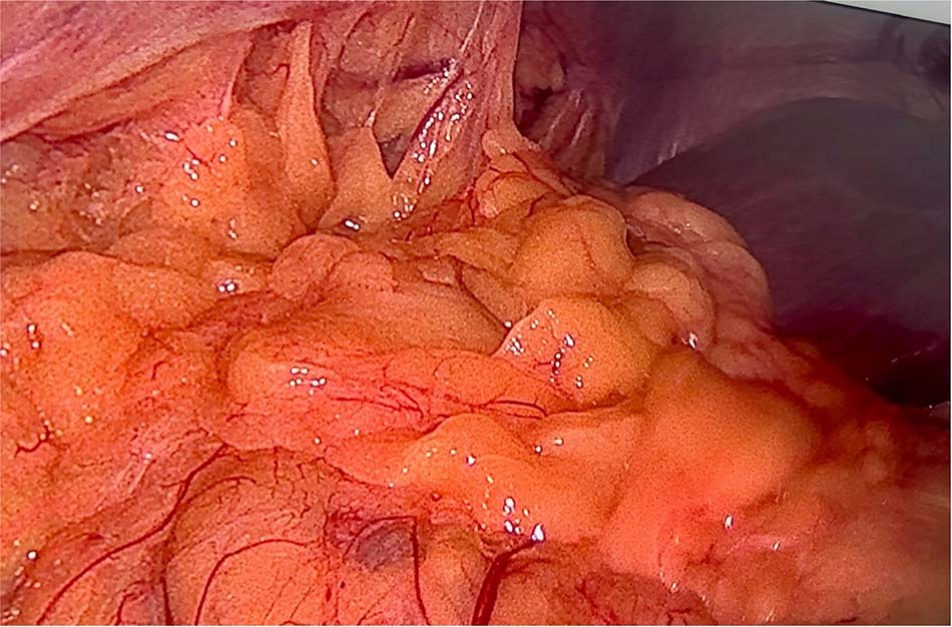
Intraoperative image reveals a right hemidiaphragm defect with omental protrusion and hepatic colon adhesion.

**Figure 4:**
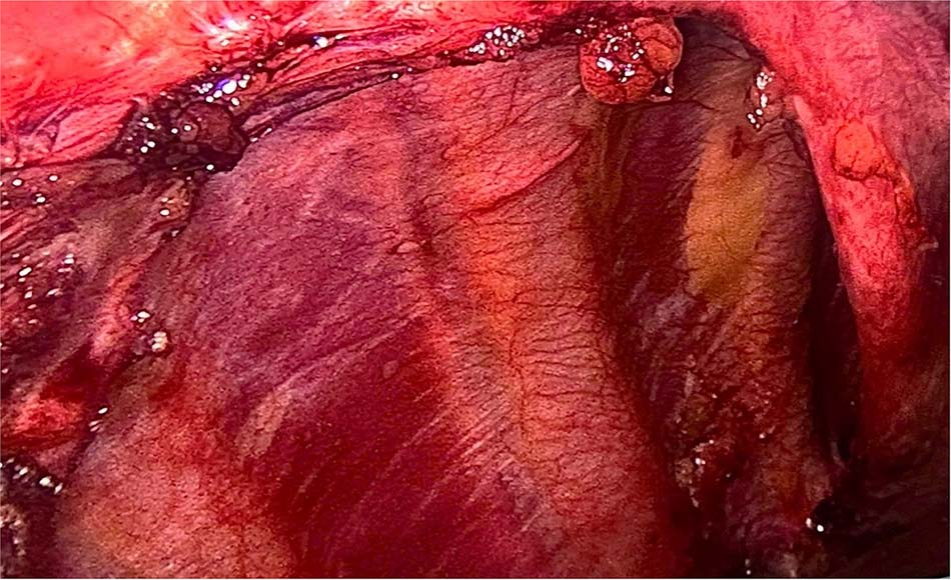
After releasing, the herniated components and reducing them back into the abdominal cavity.

**Figure 5:**
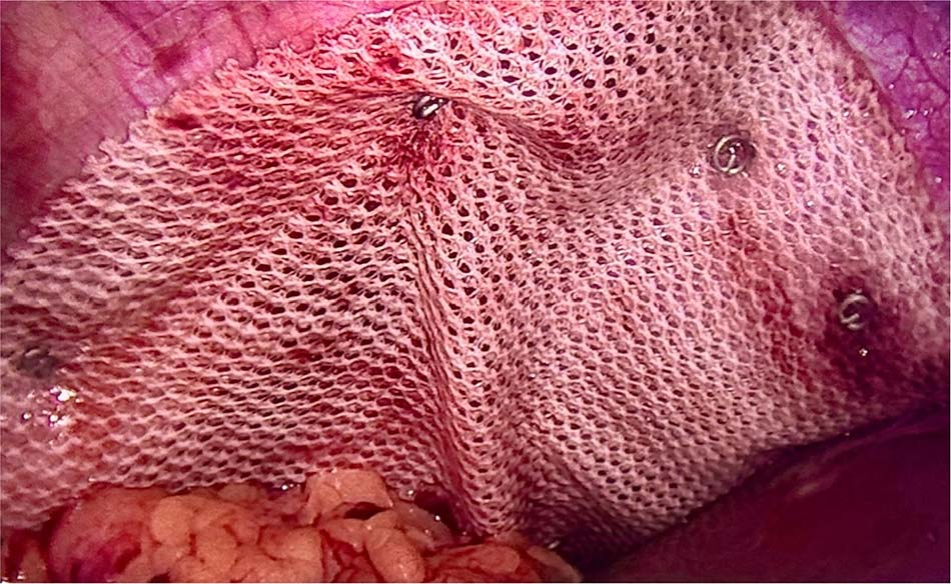
Intraoperative image demonstrating composite mesh placement over the diaphragmatic defect.

**Figure 6:**
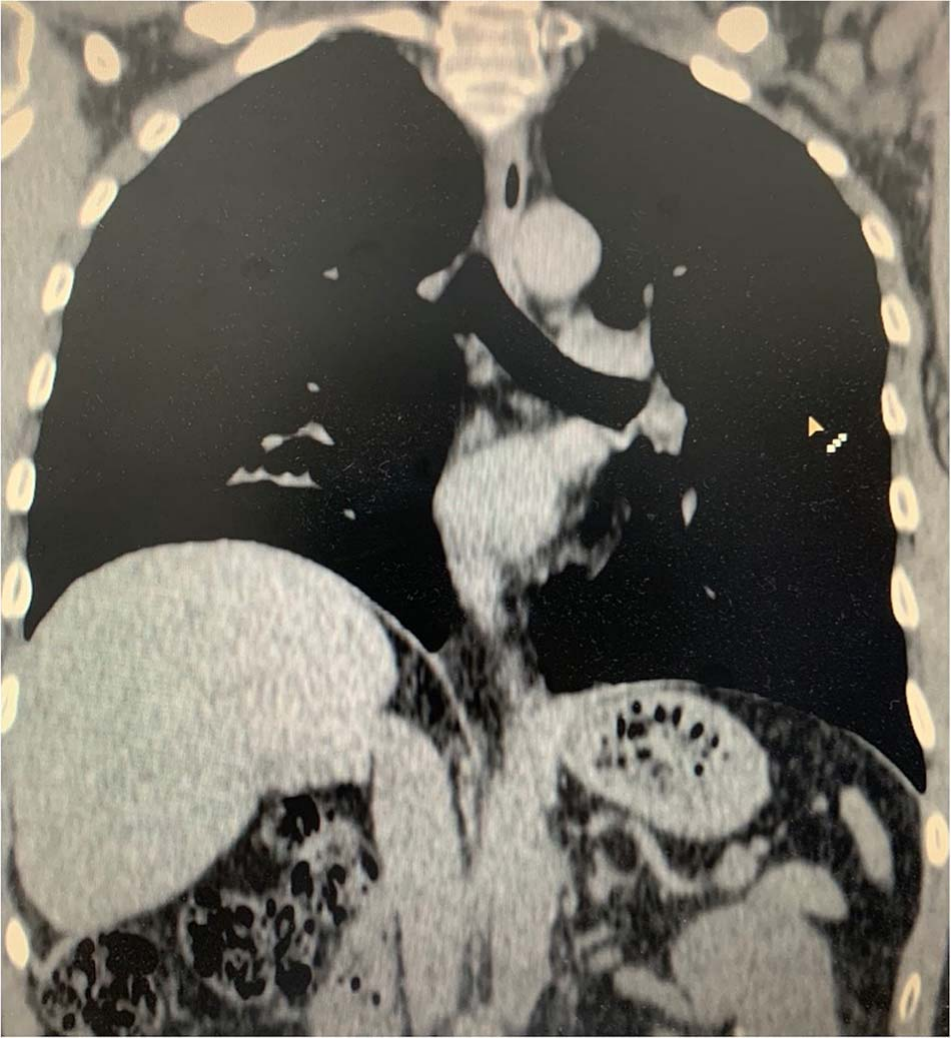
Chest CT image for re-evaluation after 5 months.

A right diaphragmatic hernia secondary to blunt abdominal trauma was diagnosed. The patient underwent laparoscopic right diaphragmatic hernia repair with mesh hernioplasty. Intraoperatively, a large defect in the right hemidiaphragm allowed omentum and hepatic flexure herniation with dense adhesions (Fig. 3). Due to the chronicity and size of the defect, complete primary closure was not feasible (Fig. 4). The defect was subsequently reinforced with a 20 × 15 cm intraperitoneal composite mesh fixed with tackers to ensure robust repair (Fig. 5). The surgical intervention was successful. The patient was discharged on Day 3, and a 5-month follow-up CT confirmed correct mesh positioning without complications (Fig. 6).

## Discussion

TDHs are uncommon, complicating <1% of all traumatic patients, resulting from blunt or penetrating trauma that disrupts the diaphragm [4, 6]. Notably, left-sided diaphragmatic hernias account for 50%–80% of cases, while right-sided hernias comprise 12%–40%, and double-sided hernias are rare. This asymmetrical distribution is attributed to the anatomical protective mechanism of the liver's bare area on the right side of the diaphragm, which helps dissipate traumatic forces and potentially reduces the likelihood of diaphragmatic rupture [4, 5, 7].

Diaphragmatic tears represent a diagnostic challenge, with up to 70% of cases initially overlooked during clinical assessment. Patient outcomes vary, ranging from complete asymptomatic resolution to chronic abdominal complaints or acute intestinal obstruction requiring urgent intervention [3, 5]. The clinical presentation is frequently nonspecific and may mimic other gastrointestinal conditions such as peptic ulcer disease or cholelithiasis. In case of incarceration, patients may exhibit symptoms including intense chest or abdominal pain, dyspnea, respiratory compromise, fever, tachycardia, and dehydration. Notably, some individuals remain asymptomatic for years, with only vague upper abdominal or lower thoracic discomfort [3–5, 7]. In the present case, a 41-year-old male with a remote history of right-sided thoracic trauma developed subtle epigastric pain radiating to the right upper quadrant and intermittent dyspnea. The symptoms were initially misattributed to gastritis, leading to ineffective self-medication. A thorough retrospective history revealed prior blunt chest trauma from a motorcycle accident two decades earlier—a pivotal detail that facilitated the diagnosis. This case underscores the critical importance of comprehensive history-taking and reinforces the necessity for early professional evaluation of persistent or atypical symptoms to prevent misdiagnosis and delayed treatment.

Radiological imaging is fundamental in diagnosing diaphragmatic hernias, including chest radiography, ultrasonography, MRI, and particularly CT [6]. While chest radiographs are valuable in acute settings, their utility in chronic presentations is debated [3]. CT has notably enhanced diagnostic accuracy, offering high sensitivity (70%–100%) and specificity (75%–100%), with slight differences between right- and left-sided hernias. Moreover, CT facilitates comprehensive assessment of hernia characteristics, including defect size, contents, associated complications, and anatomical relationships [5, 8].

Surgical intervention remains the cornerstone of diaphragmatic hernia management, with traditional approaches including laparotomy and thoracotomy, often selected based on the surgeon’s expertise. Laparoscopy has provided a less invasive alternative, offering advantages such as reduced hospital stay, faster recovery, decreased perioperative pain, and superior cosmetic outcomes. However, these benefits are contingent upon surgical proficiency and a thorough understanding of hernia anatomy. When primary closure is unfeasible due to large defect size, mesh reinforcement becomes necessary [5, 6]. In the present case, laparoscopic repair was performed, and a 20 × 15 cm polytetrafluoroethylene mesh was utilized to restore diaphragmatic integrity.

## Conclusion

TDH is a rare clinical condition, particularly on the right side. Its presentation is often nonspecific, delaying timely diagnosis. Patients may remain asymptomatic for years or exhibit subtle symptoms easily mistaken for other disorders. Accurate diagnosis requires a thorough clinical approach, emphasizing detailed history-taking and comprehensive physical examination. Evaluation of persistent or atypical symptoms by a medical professional is essential, as delayed diagnosis due to self-treatment may result in serious complications.

## Data Availability

Data are contained within the article. Data sharing does not apply to this article.
